# Bilateral Lumbar Hernias Following Spine Surgery: A Case Report and Laparoscopic Transabdominal Repair

**DOI:** 10.1155/2020/8859106

**Published:** 2020-07-31

**Authors:** Marc Rafols, Daniel Bergholz, Anthony Andreoni, Chase Knickerbocker, Jennifer Davies, Robert A. Grossman

**Affiliations:** ^1^Department of General Surgery, Mount Sinai Medical Center, Miami Beach, FL 33140, USA; ^2^University of Miami Miller School of Medicine, Miami, FL 33136, USA; ^3^Department of General Surgery, Naples Community Hospital, Naples, FL 34102, USA

## Abstract

Lumbar hernias are rare abdominal wall defects. Fewer than 400 cases have been reported in the literature and account for 2% of all abdominal wall hernias. Lumbar hernias are divided into Grynfelt-Lesshaft or Petit hernias. The former are hernia defects through the superior lumbar triangle, while the latter are defects of the inferior lumbar triangle. Primary lumbar hernias are further subdivided into congenital or acquired hernias and can further be classified as either primary or secondary. Secondary hernias occur after previous flank surgeries, iatrogenic muscular disruption, infection, or trauma. We review a rare presentation of metachronous symptomatic bilateral secondary acquired lumbar hernia following spine surgery. A successful laparoscopic transabdominal lumbar hernia repair with extraperitoneal mesh placement was performed, with resolution of the hernia symptoms. An extensive literature review regarding lumbar hernia and different types of repairs was performed.

## 1. Introduction

**Figure 1:**
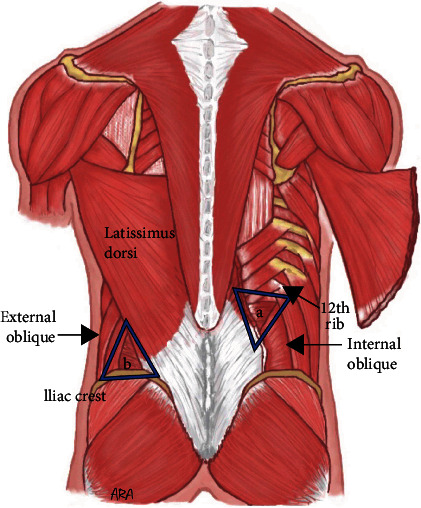
(a) The superior lumbar triangle bordered by the 12th rib, internal oblique, and quadratus (not visualized, deep to the thoracolumbar aponeurosis) and (b) the inferior triangle bordered by the iliac crest, external oblique, and latissimus dorsi.

Lumbar hernias are a very rare abdominal wall defect [1]. Fewer than 400 cases have been reported in the literature and account for 2% of all abdominal wall hernias [2, 3]. Lumbar hernias can be categorized by location and etiology into the Grynfelt-Lesshaft and Petit hernias. Grynfelt-Lesshaft are hernia defects through the superior lumbar triangle, defined as the area between the internal oblique, quadratus lumborum, the 12th rib and serratus posterior, external oblique and latissimus muscle, and the transversus abdominis aponeurosis [4]. Petit hernias are defects of the inferior lumbar triangle, defined as the area between the external oblique muscle, latissimus dorsi muscle, iliac crest, superficial fascia, and the internal oblique [5, 6] (Figure 1). The larger surface area size of the superior triangle compared to the inferior triangle is believed to be the reason why Grynfelt-Lesshaft hernias are much more common than Petit hernias [7].

Lumbar hernias are further subdivided into congenital and acquired, which make up 20% and 80% of lumbar hernias, respectively. Acquired lumbar hernias can be either primary or secondary [4]. Primary acquired lumbar hernias are more common, comprising 55% of all lumbar hernias [4]. Risk factors for developing a primary hernia are obesity, connective tissue disease, poor nutritional status, and conditions that increase intra-abdominal pressure [8]. Secondary lumbar hernias are either iatrogenic, traumatic, or following infection or inflammation and account for 25% of acquired lumbar hernias [9]. Most incisional lumbar hernias have been described to occur following retroperitoneal nephrectomies, retroperitoneal abdominal aortic aneurysm repairs, or latissimus dorsi flaps for breast reconstruction [10–12]. Very few cases of lumbar hernia have been reported following spinal fusion and, to the authors' knowledge, no case has been reported of a bilateral lumbar hernia following multiple spine surgeries [13–15]. Most reported bilateral lumbar hernias are congenital and are associated with the diffuse type which presents as aplasia of the lumbar muscles [16]. Acquired bilateral lumbar hernias have rarely been reported. Fokou et al. describe a spontaneous synchronous bilateral hernia that presented with strangulated small bowel [17]. Bilateral lumbar hernias following trauma have also been described [18].

Lumbar hernias following surgery may be difficult to differentiate from abdominal wall atrophy secondary to denervation; presentation and symptoms may be similar, and both may be treated in a similar fashion [19].

Preoperative CT imaging is essential to confirm the diagnosis, for preoperative planning, and to rule out any incarcerated abdominal organs. Although strangulation of abdominal contents has been described in about 30 cases, one must always have a degree of suspicion when faced with a nonreducible lumbar mass [20]. The risk for incarceration is reported to be lower than 10% due to the typical wide neck of the defect and its anatomic location [21]. However, anatomic radiologic studies suggest that the musculoaponeurotic tunnel existing between the superior and inferior lumbar triangles is the cause of incarceration or strangulation when present [22].

Once a lumbar hernia is confirmed, surgical repair is the standard of care [8]. Several different techniques to repairing lumbar hernias have been described. Both open and laparoscopic are accepted approaches, and each has advantages and disadvantages. In our case, we decided to approach the lumbar hernia via the transabdominal approach, with extraperitoneal mesh placement and closure of the peritoneum over the mesh.

## 2. Case Report

**Figure 2:**
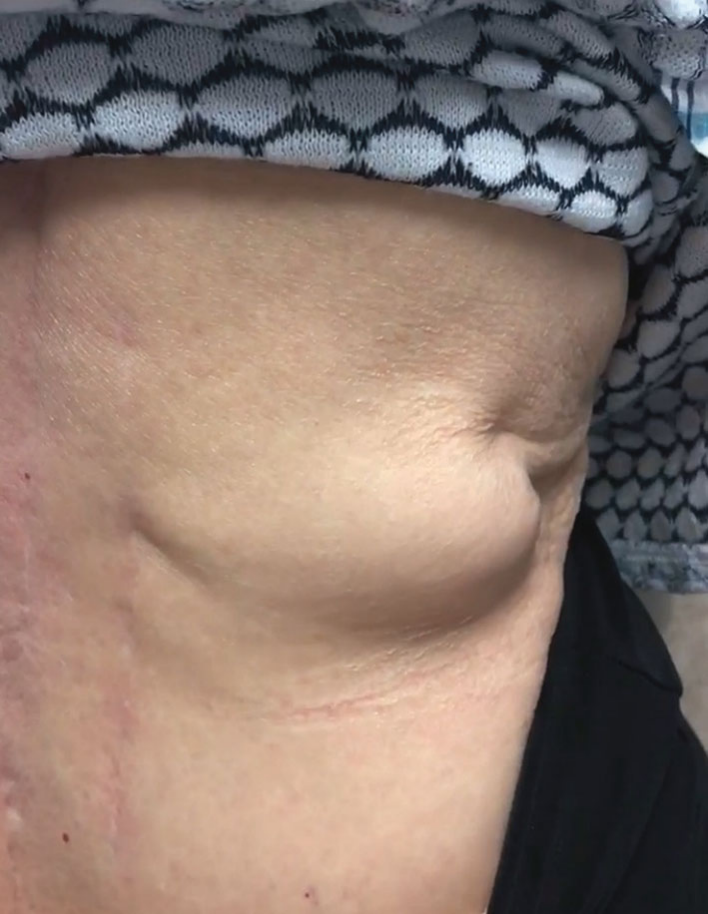
Right superior lumbar hernia bulge while performing a Valsalva maneuver.

An 83-year-old female presented with a right flank soft tissue mass that had been progressively increasing in size. The mass was associated with worsening right flank pain radiating to her right chest that was exacerbated by getting up from the sitting position, abduction of the right arm, or turning in bed. On physical examination, a reducible right flank bulge was noted to increase in size when performing a Valsalva maneuver or standing from a seated position (Figure 2). The patient had a past medical history of rheumatoid arthritis, osteoporosis, chronic kidney disease, hypothyroidism, and an extensive history of chronic back pain. She had a past surgical history of bilateral mastectomy without reconstruction, total hysterectomy and oophorectomy, total colectomy with ileocolic anastomosis for colonic inertia, and several spine surgeries. The spinal surgeries included a T12-L2 fusion for postlaminectomy syndrome and lumbar spine stenosis in 2012, which were preformed from a combined lateral and posterior approach. She also has a history of a contralateral left-sided open lumbar hernia repair with mesh performed at an outside hospital two years prior to presentation. A plain abdominal CT scan demonstrated a right lumbar hernia in the superior lumbar triangle containing retroperitoneal fat with no sign of incarceration or ischemia. We decided to perform a transabdominal laparoscopic hernia repair with extraperitoneal mesh placement.

## 3. Surgical Repair

**Figure 3:**
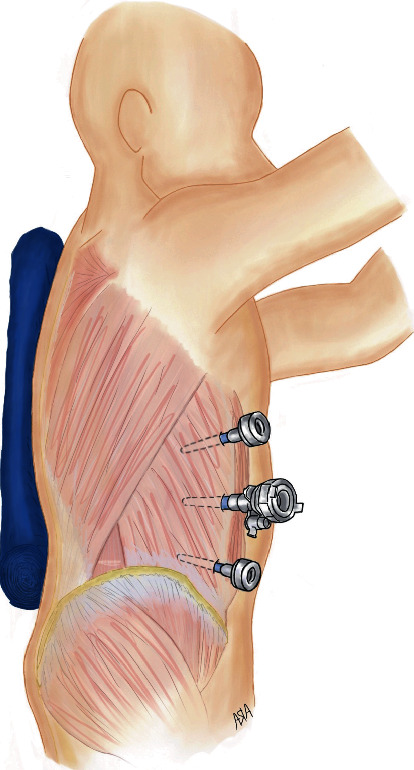
Left lateral decubitus positioning with trocar placement. 5 mm trocars were inserted in the right subcostal area and in the right lower quadrant. The 12 mm trocar was placed along the midclavicular line.

The patient was placed in the left lateral decubitus position using a bean bag for support. Due to the patient's previous surgeries, the abdomen was entered with a Veress needle in the right upper quadrant and pneumoperitoneum was obtained. A 5 mm trocar was used to enter the abdomen in the right upper quadrant. Additional 5 mm and 12 mm trocars were inserted in the right subcostal line (Figure 3). The lumbar hernia defect was identified, and the retroperitoneum was incised anterior to the defect. Retroperitoneal fat was reduced from the defect, which was measured to be approximately 2 cm. The defect was closed with a number 0 self-locking suture. A 4 × 4 cm piece of uncoated polypropylene mesh was used to cover the defect and was fastened in place with 5 mm absorbable laparoscopic tacks. Tacks were placed judiciously over the posterior mesh border to avoid injury to the iliohypogastric, ilioinguinal, and genitofemoral nerves. The peritoneum was then closed over the mesh with a 2-0 self-locking suture (see video Supplemental digital content 1, which highlights the laparoscopic transabdominal lumbar hernia repair).

**Figure 4:**
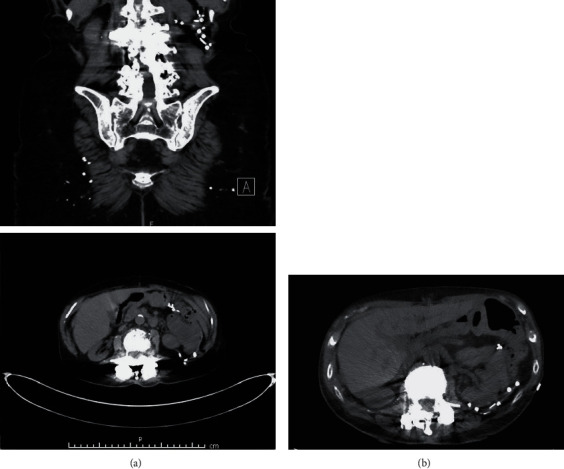
(a) Preoperative CT scan demonstrating a superior lumbar hernia defect containing retroperitoneal fat. (b) Postoperative imaging 3 months after surgery.

The patient recovered well and was discharged on postoperative day one. Her postoperative course was significant for development of a small postoperative hematoma within the hernia space, which resolved spontaneously. Her right-sided flank pain was resolved. Pre- and postoperative CT imaging can be seen in Figures 4(a) and 4(b).

## 4. Conclusion

Acquired lumbar hernias are extremely rare. Bilateral lumbar hernias following spinal fusion surgery have not been described well in the literature, and as such, there are no clear guidelines for their management. Each lumbar hernia needs to be approached in an individualized manner. In our scenario, advanced age, multiple comorbidities, and previous abdominal surgeries influenced the decision to perform a transabdominal laparoscopic lumbar hernia repair with retroperitoneal mesh placement. Laparoscopic lumbar hernia repair is a safe approach that is associated with less postoperative pain, decreased hospital stay, and a quick recovery.

## Supplementary Materials

Supplementary Materials Supplemental Video 1: this video demonstrates a laparoscopic transabdominal lumbar hernia repair with extraperitoneal mesh placement.
